# Anstieg der natürlichen Hörschwelle nach Cochleaimplantatversorgung

**DOI:** 10.1007/s00106-023-01398-4

**Published:** 2024-01-05

**Authors:** Nathalie Moermans, Holger Sudhoff, Ingo Todt

**Affiliations:** Klinik für Hals-Nasen-Ohrenheilkunde, Kopf- und Halschirurgie, Medizinische Fakultät OWL, Universität Bielefeld, Klinikum Bielefeld Mitte, Teutoburger Str. 50, 33604 Bielefeld, Deutschland

**Keywords:** ISSNHL, Auditorische Neuropathie, Regeneration, Höranstieg, Idiopathic sudden sensorineural hearing loss, Auditory Neuropathy, Regeneration, Hearing increase

## Abstract

Mehr als 5 % der Weltbevölkerung leiden an einem behindernden Hörverlust. Bei unklarer Ursache des Hörverlusts bezeichnet man dies als „idiopathic sudden sensorineural hearing loss“ (ISSNHL). Nach ausbleibendem Erfolg der Standardtherapie wird in aller Regel die Verwendung von Hörgeräten oder ein Cochleaimplantat (CI) empfohlen. In diesem Fall wurde ein 55-jähriger Patient mit ISSNHL und erfolgloser konservativer Therapie mit einer Cochleaimplantation behandelt. Rund 1 Jahr nach Implantation und 7 Jahre nach dem Hörsturz wurde anhand von subjektiven Messungen eine weitestgehende Wiederherstellung der Hörschwelle festgestellt.

## Abstract (en)

More than 5% of the world’s population suffers from disabling hearing loss. If the cause of hearing loss is unclear, it is referred to as idiopathic sudden sensorineural hearing loss (ISSNHL). After failure of standard treatment, the use of hearing aids or a cochlear implant is generally recommended. In this case, a 55-year-old patient was treated with cochlear implantation (CI) after ISSNHL and unsuccessful conservative therapy. Approximately 1 year after implantation and 7 years after the sudden hearing loss, subjective measurements revealed restoration of the hearing threshold.

Laut der Weltgesundheitsorganisation (WHO) leiden 430 Mio. Menschen (mehr als 5 % der Weltbevölkerung) an einem behindernden Hörverlust, der einer Rehabilitation bedarf [1]. Oft kann die Ursache des Hörverlusts trotz umfänglicher Diagnostik jedoch nicht festgestellt werden, was als „idiopathic sudden sensorineural hearing loss“ (ISSNHL) bezeichnet wird. ISSNHL ist definiert als plötzlich (innerhalb von 72 h) auftretender, meist einseitiger Hörverlust cochlearen Ursprungs von mindestens 30 dB in drei aufeinanderfolgenden Frequenzen mit unbekannter Ursache [2]. Bei fehlendem Ansprechen auf die konservative Therapie ist die Verwendung von Hörgeräten, ggf. – je nach Ausprägung des Hörverlusts – die Implantation eines Cochleaimplantats (CI) indiziert. Eine verspätete spontane Erholung des Hörvermögens ist selten. Wir stellen einen Patienten mit einseitigem ISSNHL vor, der mit einem Cochleaimplantat behandelt wurde und 1 Jahr nach der Implantation einen signifikanten Anstieg der natürlichen Hörschwelle entwickelte.

## Falldarstellung

### Anamnese und Befund

**Abb. 1:**
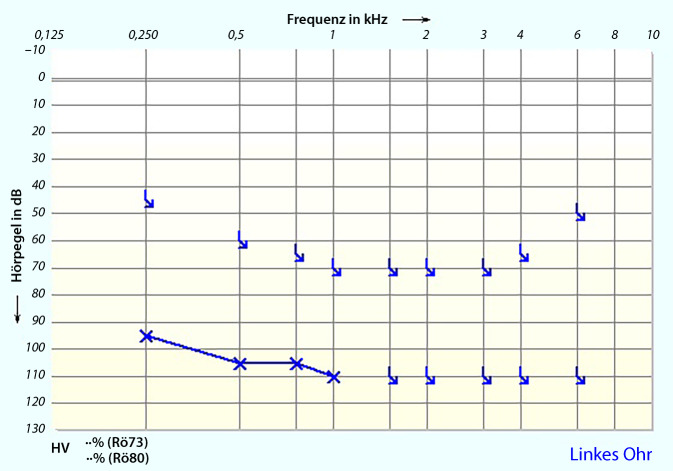
Audiogramm des linken Ohrs in der Akutsituation (2013)

**Abb. 2:**
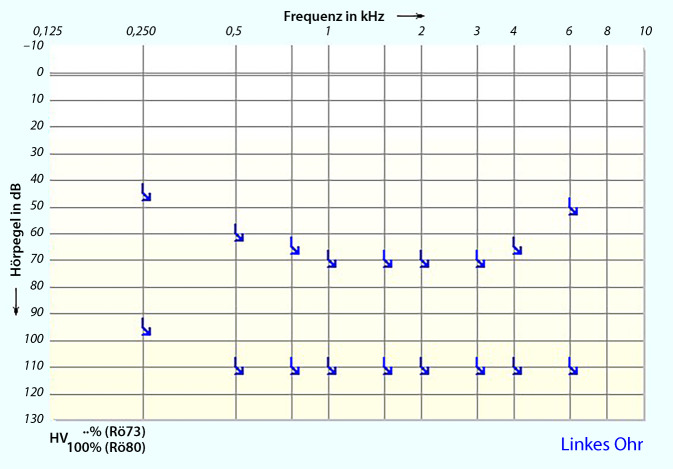
Audiogramm des linken Ohrs nach 6 Jahren (2019)

Ein 55-jähriger Mann stellte sich mit akuter linksseitigen, an Taubheit grenzender Schwerhörigkeit vor (Abb. 1). Anamnestisch gab er intermittierenden Schwindel, Tinnitus links sowie Taubheitsgefühl in der linken Gesichtshälfte an. Komorbiditäten des Patienten waren eine arterielle Hypertonie und Diabetes mellitus bei einem Zustand nach einer Pankreas-Teilresektion. Zudem erlitt er einen Herzinfarkt sowie zweimal einen Apoplex mit Hemiplegie links. Mittels MRT wurden ein Tumor im Kleinhirnbrückenwinkel sowie im übrigen Neurokranium sowie ein frischer Infarkt ausgeschlossen. Nach erfolgloser Steroidgabe wurde im Jahr 2014 eine Tympanoskopie mit Abdeckung des ovalen und runden Fensters durchgeführt, ohne Verbesserung der Hörschwelle. Mittels Hörschwellenbestimmung (PTA) und objektiver Verfahren waren fünf Jahre später keine Potenziale auf dem linken Ohr ableitbar (Abb. 2 und 3), was im September 2019 zur Entscheidung für eine Cochleaimplantation führte. In den darauffolgenden Jahren war der Patient zufrieden mit dem Implantat und erreichte eine Sprachverständlichkeit von 80 % im Freiburger Einsilbertest mit dem Implantat. Trotzdem klagte er im Mai 2021 über Schmerzen und Schwindel bei der Verwendung des Cochleaimplantats (CI). Er berichtete von ca. fünf Episoden mit Schwankschwindel, welche 20 min anhielten. Auf diesem Grund verwandte er das Implantat nur gelegentlich. Die zu diesem Zeitpunkt durchgeführte Hörschwellenbestimmungen (PTA) zeigten einen Anstieg der Hörschwelle auf der linken Seite von 25 dB von 250 Hz bis 1 kHz mit Knochenleitung (Abb. 4), bestätigt durch objektive Messergebnisse (Abb. 5). Das Tragen des CI-Audioprozessors durch den Patienten wird derzeit abgelehnt.

**Abb. 3:**
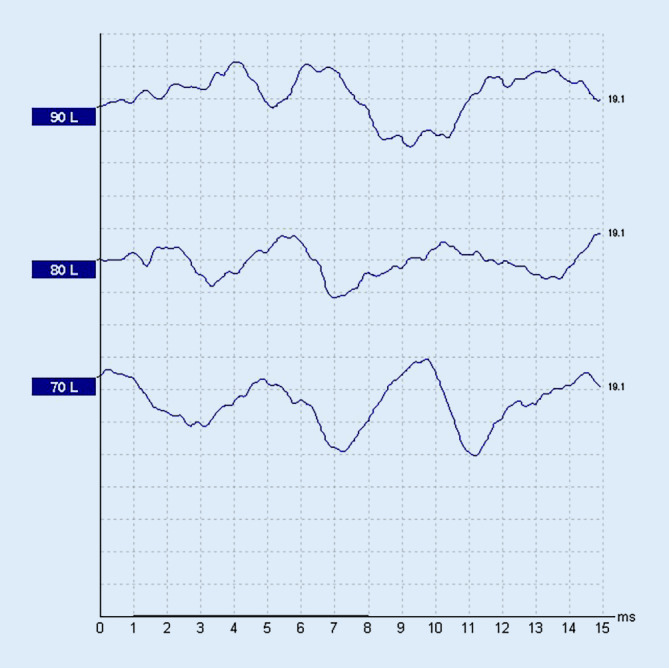
BERA des linken Ohrs vor Cochleaimplantation (2019)

**Abb. 4:**
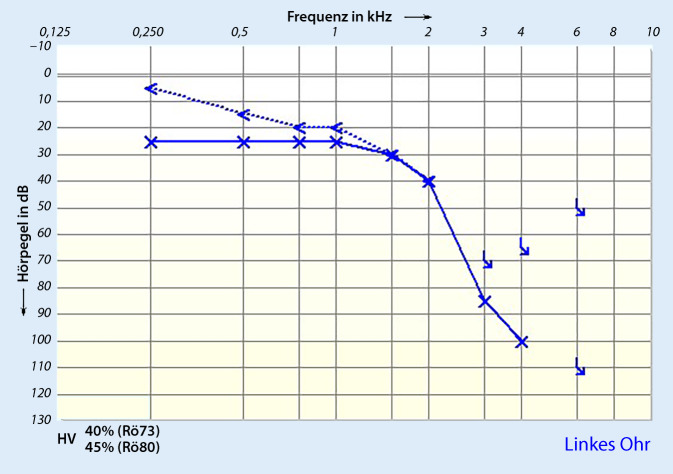
Audiogramm des linken Ohrs gemessen ohne Cochleaimplantat (11/2021)

**Abb. 5:**
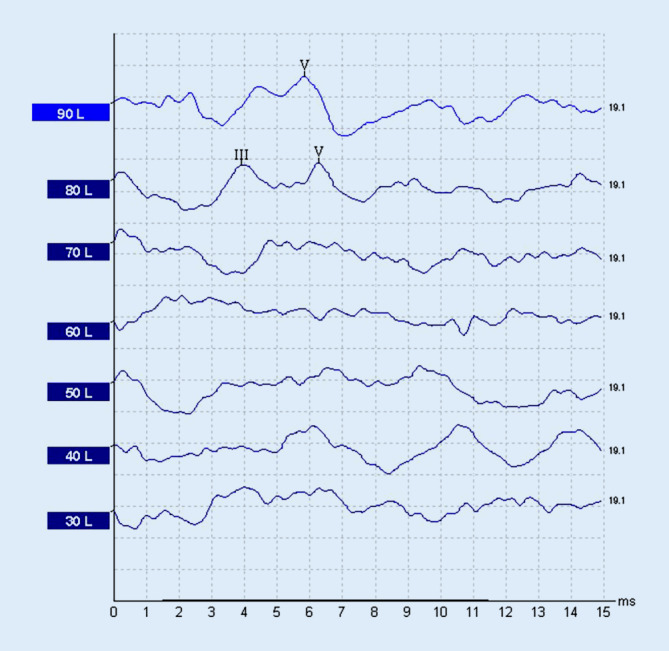
BERA des linken Ohrs gemessen ohne Cochleaimplantat (11/2021)

## Diskussion

Die Literatur besagt, dass die Hörschwelle bei 47–63 % der Patienten mit ISSNHL im Laufe von zwei Wochen auf natürliche Weise ansteigt [3]. Bei Patienten, die wegen ISSNHL behandelt werden, erholt sich die Hörschwelle bei 21–28 % vollständig [4]. Obwohl die meisten Patienten ein Ansprechen auf die Behandlung in den ersten Wochen zeigen, beschreibt eine retrospektive Studie, dass sich die Genesung bei mehr als 20 % der Patienten um mindestens 2 Monate nach Therapieende (mit systemischer Kortikosteroide und sekundärer intratympanaler Kortikoidinjektion) verzögert. Es ist daher wichtig, Patienten mit anfänglich geringem Ansprechen auf die Therapie einen ausreichend langen Beobachtungszeitraum anzubieten. Die American Academy of Otolaryngology – Head and Neck Surgery Guidelines empfehlen einen Zeitraum von bis zu 6 Monaten [3].

Interessant in unserem Fall ist, dass sich das Gehör nach mehr als 7 Jahren erholte. Es ist unklar, was die plötzliche Verbesserung hier verursacht hat, zumal eine Cochleaimplantatversorgung erfolgte. Als positiven prognostischen Faktor für eine verzögerte Genesung konnten Na et al. [3] neben der Schwere des Hörverlusts auch das höhere Alter und das schlechte Ansprechen auf eine Akuttherapie nachweisen. Die Autoren nehmen an, dass die verzögerte Heilung kein natürlicher Prozess ist, sondern eine späte Reaktion auf die verabreichte Kortikosteroidtherapie sei. Sie stützen sich dabei auf eine Studie, die zeigte, dass Patienten, die keine Sekundärtherapie mit intratympanaler Injektion erhielten, signifikant seltener eine verzögerte Heilung aufwiesen [5]. Allerdings hatte unser Patient seit mehreren Jahren keine Kortisontherapie erhalten.

Xiong et al. [6] beschreiben, dass eine Restitutio ad integrum nach ISSNHL bei Patienten unter 30 Jahren sowie bei Patienten ohne Schwindel wahrscheinlicher ist. Komorbiditäten wie arterielle Hypertension, Diabetes mellitus, Dyslipidämie und Schilddrüsenerkrankungen wurden als negative prognostische Faktoren beschrieben. In derselben Studie wurde ein größerer Anstieg der Hörschwelle in den tiefen Frequenzen beobachtet. Die Autoren erklären dies damit, dass sich apikale Haarzellen wahrscheinlicher erholen als basale Haarzellen. Auch bei unserem Patienten findet sich der größte Anstieg im Mittel- bis Tieftonbereich. Anzumerken ist jedoch hier, dass das Defizit in der Hörschwelle im Hochtonbereich ggf. auf ein intracochleäres Trauma, bedingt durch die CI-Versorgung, zu erklären sein könnte.

Seo et al. [4] entdeckten einen möglichen Zusammenhang zwischen cochleärer Synaptopathie und vollständiger Genesung nach ISSNHL. Bei cochleärer Synaptopathie nimmt die Anzahl der Bandsynapsen zwischen den inneren Haarzellen und dem Hörnerv ab. Dies führt zu einer Beeinträchtigung des Sprachverständnisses, trotz eines normalen Audiogramms (Haarzellfunktion/OAE). Bei Patienten mit vollständiger Genesung nach ISSNHL stellte man bei Hirnstammaudiometrie, im Vergleich zur gesunden Seite, eine kleinere Amplitude der I‑Welle fest. Außerdem wurde anhand von 2 Fragebögen festgestellt, dass 26,3 und 36,8 % der Patienten (mit normalem Audiogramm) leichte Hörprobleme angaben. Diese beiden Feststellungen könnten darauf hinweisen, dass Patienten nach vollständiger Genesung von ISSNHL immer noch eine cochleäre Synaptopathie haben. Laut Autoren könnte daher eine Beteiligung der cochleären Synaptopathie an der Pathogenese von ISSNHL bestehen. Allerdings sind Untersuchungen mit einer größeren Anzahl von Patienten erforderlich. Möglich ist, dass eine cochleäre Synaptopathie im klinischen Verlauf unseres Patienten eine Rolle spielt. Hinweisend könnte sein, dass nach Cochleaimplantation und Anstieg der Hörschwelle (11/2021) mittels BERA nur partiell Potenziale ableitbar sind (Abb. 5).

Raymond et al. [7] haben im Jahr 2022 einen Fall beschrieben, bei dem die Hörschwelle einer Patientin mit ausgeprägtem Hörverlust aufgrund eines Morbus Menière sich direkt postoperativ nach einer Cochleaimplantation teilweise erholte. Diese Hörverbesserung war sechs Monate später immer noch mittels einer Hörschwellenbestimmung (PTA) nachzuweisen. Die Autoren besprechen drei mögliche Theorien: natürliche Fluktuation des Hörvermögens bei Morbus Menière, ein Effekt der Kortikosteroide oder Druckänderungen der Wanderwelle durch die in der Scala tympani befindliche CI-Elektrode.

Ein ähnlicher Fall wurde von Ortmann et al. [8] beschrieben. Im Jahr 2008 stellte sich eine 53-jährige Patientin mit hochgradiger Innenohrschwerhörigkeit sowie Tinnitus rechts vor. Einen Monat nach Beendung der Therapie mit intratympanalen Injektionen wurde mittels Audiometrie (PTA) ein Anstieg der Hörschwelle von 20 bis 30 dB gemessen. Nach ca. einem Jahr hatte sich das Hörvermögen auf dem rechten Ohr vollständig erholt, welches mittels Audiometrie (PTA) bestätigt wurde. Die Autoren diskutieren, dass es sich also um eine reversible Schädigung im Innenohr als Ursache der initialen Hörminderung handeln muss. Sie vermuten einen möglichen Defekt in der Homöostase der Cochlea und damit eine Änderung des endocochleären Potenzials. Was unklar bleibt, ist, was dazu geführt hat, dass das Hörvermögen sich erst nach 9 Monaten erholt hat. In unserem Fall ist die Zeitspanne sogar noch länger (Steroidgabe im Jahr 2013, Cochleaimplantation 2019, Anstieg der Hörschwelle 2021).

Wie beschrieben, weist unser Patient zweimal einen Apoplex in der Vorgeschichte auf. Obwohl in einer Metaanalyse ein Zusammenhang zwischen Schlaganfall und Hörverlust gezeigt wurde [9], gehen wir davon aus, dass zwischen diesen Apoplexien (vor 2013) und dem Hörverlust (Ende 2013) kein direkter zeitlicher Zusammenhang besteht. In unserem Fall konnte 2013 mittels MRT nur ein alter lakunärer Defekt rechts dargestellt werden. Aufgrund der Vorgeschichte unseres Patienten wäre es jedoch nicht auszuschließen, dass ein kleines ischämisches Ereignis aufgetreten ist, welches zu dem akuten Hörverlust geführt hat. Ggf. könnte sich ein solcher Mikroinfarkt der MRT-Auflösung entzogen haben.

## Fazit für die Praxis

- Ein verzögerter Anstieg der Hörschwelle nach ISSNHL ist nicht ungewöhnlich.
- Außergewöhnlich in unserem Fall ist jedoch, dass eine Erholung der Hörschwelle erst nach 7 Jahren eingetreten ist.
- Des Weiteren hat sich das Hörvermögen erholt, nachdem eine Cochleaimplantation erfolgte.
- Der zugrunde liegende Mechanismus ist unklar.

## References

[1] https://www.who.int/news-room/fact-sheets/detail/deafness-andhearing-loss. Zugegriffen: 10. Nov. 2022

[2] Chandrasekhar SS, Do TBS, Schwartz SR, Bontempo LJ, Faucett EA, Finestone SA, Hollingsworth DB, Kelley DM, Kmucha ST, Moonis G, Poling GL, Roberts JK, Stachler RJ, Zeitler DM, Corrigan MD, Nnacheta LC, Satterfield L (2019). Clinical Practice Guideline: Sudden Hearing Loss (Update). Otolaryngol Head Neck Surg. 2019. Aug.

[3] Na G, Kim KW, Jung KW, Yun J, Cheong TY, Lee JM (2022) Delayed Recovery in Idiopathic Sudden Sensorineural Hearing Loss. J Clin Med 11(10):2792. 10.3390/jcm11102792. PMID: 35628918; PMCID: PMC9143329 10.3390/jcm11102792 PMC9143329 35628918

[4] Seo HW, Lee SY, Byun H, Lee SH, Chung JH (2022) Possible Existence of Cochlear Synaptopathy in Patients Completely Recovered from Idiopathic Sudden Sensorineural Hearing Loss. J Clin Med 11(3):875. 10.3390/jcm11030875. PMID: 35160326; PMCID: PMC8836441 10.3390/jcm11030875 PMC8836441 35160326

[5] Chen WT, Lee JW, Yuan CH, Chen RF (2015). Oral Steroid Treatment for Idiopathic Sudden Sensorineural Hearing Loss. Saudi Med J.

[6] Xiong W, Dai Q, Wang Y, Hou Z, Lu K, Sun X, Duan F, Wang H, Zhang D, Wang M (2022). Front Psychol.

[7] Raymond M, Strange C, Rizk H (2022). Recovery of Unaided Hearing After Cochlear Implantation in Patient With Bilateral Menière‘s Disease. Otol Neurotol. 2022 Sep 1. Epub.

[8] Ortmann AJ, Neely JG (2012). Sudden Sensorineural Hearing Loss and Delayed Complete Sudden Spontaneous Recovery. J Am Acad Audiol.

[9] Khosravipour M, Rajati F (2021) Sensorineural Hearing Loss and Risk of Stroke: a Systematic Review and Meta-Analysis. Sci Rep 11(1):11021. 10.1038/s41598-021-89695-2. PMID: 34040004; PMCID: PMC8155183 10.1038/s41598-021-89695-2 PMC8155183 34040004

